# Data on the putative role of p53 in breast cancer cell adhesion: Technical information for adhesion assay

**DOI:** 10.1016/j.dib.2016.09.038

**Published:** 2016-09-30

**Authors:** Kallirroi Voudouri, Dragana Nikitovic, Aikaterini Berdiaki, John Tsiaoussis, Dimitris Kletsas, Nikos K. Karamanos, George N. Tzanakakis

**Affiliations:** a Department of Anatomy-Histology-Embryology, School of Medicine, University of Crete, Heraklion, Greece; b Laboratory of Cell Proliferation and Ageing, Institute of Biology, National Center of Scientific Research "Demokritos", Athens, Greece; c Biochemistry, Biochemical Analysis and Matrix Pathobiology Res. Group, Laboratory of Biochemistry, Department of Chemistry, University of Patras, 26110 Patras, Greece

**Keywords:** Breast cancer cell adhesion, Fibronectin, Insulin growth factor receptor –I (IGF-IR), p53 tumor suppressor gene

## Abstract

In this data article, the potential role of p53 tumor suppressor gene (p53) on the attachment ability of MCF-7 breast cancer cells was investigated. In our main article, “IGF-I/ EGF and E2 signaling crosstalk through IGF-IR conduit point affect breast cancer cell adhesion” (K. Voudouri, D. Nikitovic, A. Berdiaki, D. Kletsas, N.K. Karamanos, G.N. Tzanakakis, 2016) [1], we describe the key role of IGF-IR in breast cancer cell adhesion onto fibronectin (FN). p53 tumor suppressor gene is a principal regulator of cancer cell proliferation. Various data have demonstrated an association between p53 and IGF-IR actions on cell growth through its’ putative regulation of IGF-IR expression. According to our performed experiments, p53 does not modify IGF-IR expression and does not affect basal MCF-7 cells adhesion onto FN. Moreover, technical details about the performance of adhesion assay onto the FN substrate were provided.

**Specifications Table**

**Table:** 

Subject area	*Biology*
More specific subject area	*Cell functions and signaling*
Type of data	*Graphs, figures*
How data was acquired	*Adhesion assay, Real time PCR, Western blot*
Data format	*Analyzed*
Experimental factors	*Fibronectin as a substrate for the adhesion assay, transfection with siRNA specific for p53*
Experimental features	*Various numbers of cells were plated onto FN to optimize cell number and adhesion time in order to design a tailor-made adhesion assay for MCF-7 cells. The attachment ability of cells and the expression of IGF-IR were assessed after transfection of cells with siRNA specific for p53 gene.*
Data source location	*Department of Anatomy- Histology- Embryology, School of Medicine, University of Crete*
Data accessibility	*Data are provided with this article*

**Value of the data**

• There is an established connection between p53 and IGF-IR activities in cancer; specifically p53 mutant forms are known to enhance *IGF-IR* gene expression (2–5). The assessment of p53/IGF-IR interactions in breast cancer cell adhesion can be of value for research groups from related fields.
• These data can be compared to other scientific data addressing the connection between various tumor suppressor genes and IGF-IR expression and/or to data of this interaction in other cell lines and functions.
• These data facilitates other researchers to execute the optimum adhesion assay for the evaluation of MCF-7 cell adhesion onto FN.

## 1 Data

**Fig. 1:**
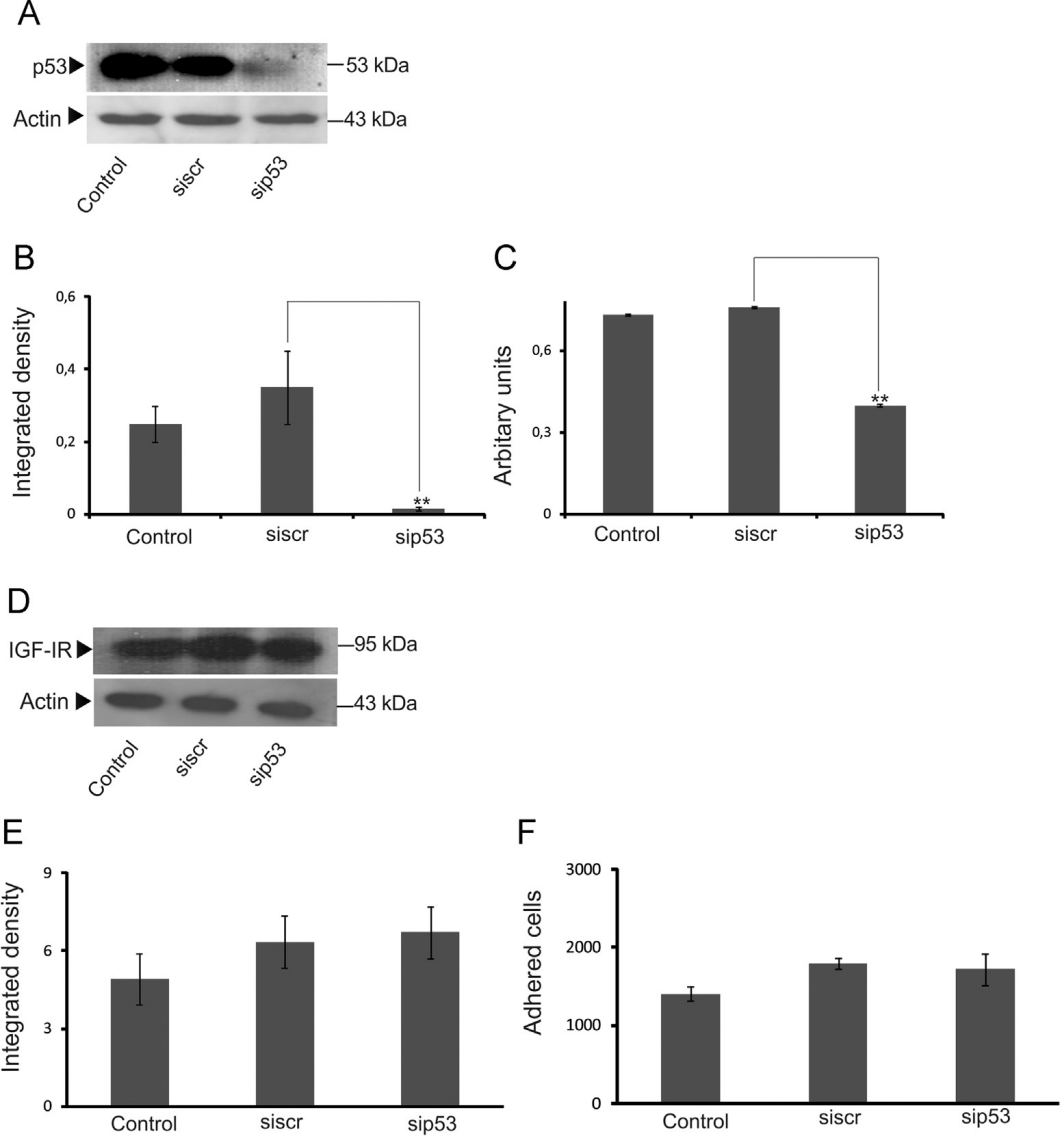
The role of p53 in breast cancer cell adhesion and IGF-IR expression. MCF-7 cells were transfected with p53 short interfering RNA (sip53), where scramble RNA (siScr) was used as a negative control. The cells were cultured for 48 h before harvesting. (A), Representative blot of p53 protein (53kDa) band is presented; (B) p53 protein bands were densitometrically analyzed and adjusted against actin. (C), p53 mRNA levels expression was verified by real time PCR; (D), MCF-7 cells were transfected with p53 short interfering RNA (si53) with the use of scramble RNA (siScr) as a negative control. The cells were cultured for 48 h before harvest and IGF-IR protein levels were verified with Western blotting; (E), p53 protein bands (p53kDa) were densitometrically analyzed and adjusted against actin. The results represent the average of three separate experiments in triplicates. Mean±SEM plotted; (F), MCF-7 cells were transfected with p53 short interfering RNA (sip53) with the use of scramble RNA (siscr) as a negative control. The cells were cultured for 48 h before harvesting and reseeding for 1 h on 96 well-plates coated with FN. The number of attached cells was determined using fluometric CyQUANT Assay Kit (Molecular Probes).

**Fig. 2:**
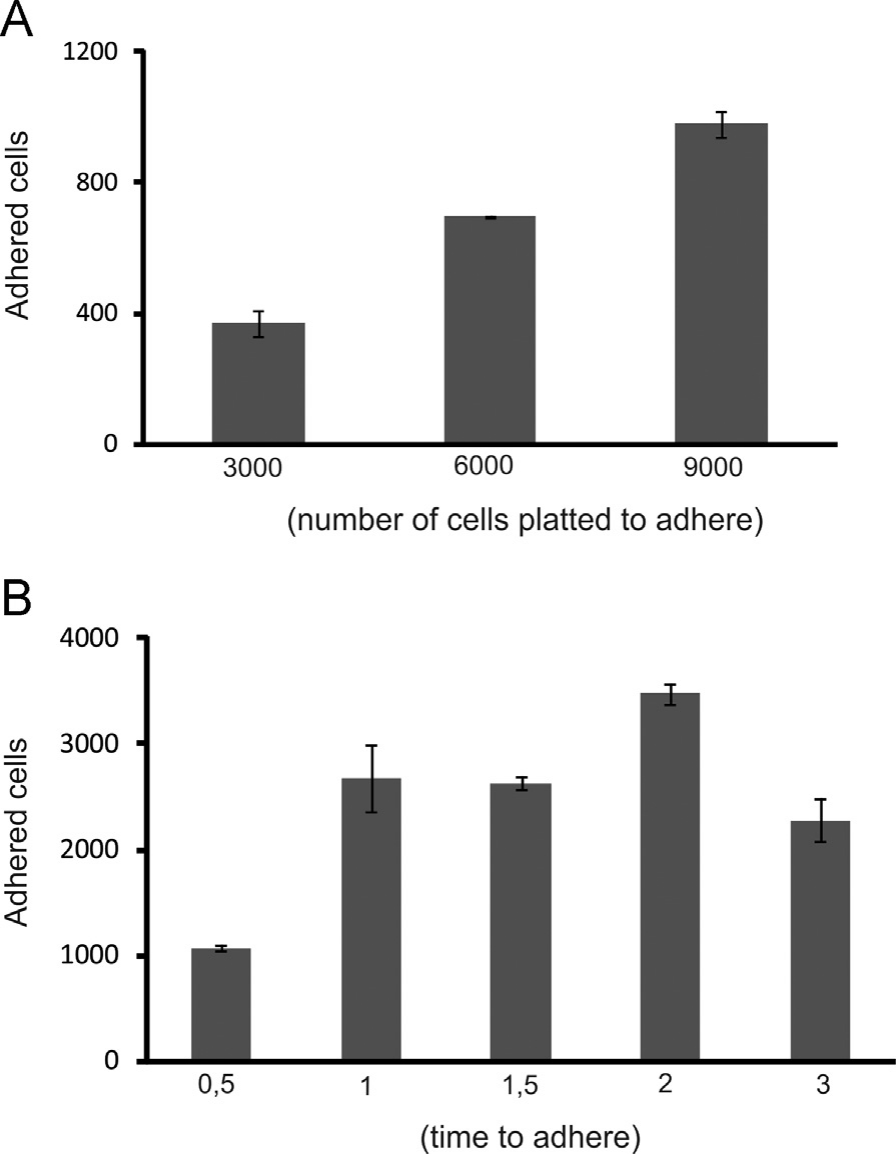
Adhesion assay optimization. MCF-7 cells were cultured for 48 h in serum- free conditions, before harvesting and reseeding on 96-well plates coated with FN for 30 min at three different concentrations (3.000, 6.000 and 9.000 cells/well) (A). 6000 cells/well seeded for 30 min, 1 h, 1,5 h, 2 h, 3 h on 96-well plates coated with FN for 1 h (B). The number of attached cells was determined by fluometric CyQUANT Assay Kit (Molecular Probes). The results represent the average of three separate experiments in triplicates. Mean±SEM plotted.

This article contains graphs presenting data on the role of p53 tumor-suppressor gene[2], [3], [4], [5] in IGF-IR expression and breast cancer cell adhesion (Fig. 1). Furthermore, technical details for the performance of the MCF-7 cells’ adhesion assay including number of plated cells and the adherence time for the MCF-7 cell adhesion protocol, are included (Fig. 2). Utilized reagents are presented in Table 1.

## 2 Experimental design, materials and methods

In order to optimize the adhesion assay protocol we utilized various cell seeding numbers and adhesion times. Cell lines and cell culture conditions are presented in [1]. In this article additional technical features of cell adhesion assay are provided.

### 2.1 Real time PCR, Western blot, adhesion assay

Adhesion assay, siRNA transfection with siRNA specific for p53, real time PCR and western blot experiments were designed to examine the role of p53 in MCF-7 FN-dependent adhesion. RNA interference, Western Blot, Real time PCR and adhesion assay protocols were described in [1]. Here, we provide extra information describing the characteristics of utilized siRNA specific for p53, p53 antibody and p53 primer (Table 1).

### 2.2 Cell adherence assay- optimization of cell number and time to adhere

The 96-well plates were coated with FN (Milipore) (5 μg/ml) as described in (1). MCF-7 cells were detached with 5 mM PBSEDTA. Cells at 3000, 6000 and 9000 cell/well were seeded onto FN coated 96-well plate. In this experiment, the cells were allowed to adhere or 30 min. The 6000 cells/well approach was chosen as having the optimum seeded cells number / adhered cell number ratio. To determine the optimum adhesion time the 6000 cells/well were allowed to adhere during 30 min, 1 h, 1,5 h, 2 h and 3 h, respectively. The number of adherent cells was determined as described in (1)

## Figures and Tables

**Table 1:** Biochemical reagents utilized for the p53 experiment.

p53 antibody (Santa Cruz Biotechnology)	Sc-126
p53 primer (VBC- Biotech)	F 5′- CGT CTG GGC TTC TTG CAT TC-3′
	R 5′- AAG ACC TGC CCT GTG CAG C-3′
p53 siRNA	5′-CAGTCTACCTCCCGCCATA-3′
	5′-GAAGAAACCACTGGATGGA-3′
