# Supplementary material for: Data on the putative role of p53 in breast cancer cell adhesion: Technical information for adhesion assay
Source: Data Brief. 2016 Sep 30;9:568–72. doi: 10.1016/j.dib.2016.09.038 (PMC5061116; doi:10.1016/j.dib.2016.09.038)
Supplement: Supplementary file 1 — Supplementary material [file mmc1.docx]

All authors declare that there is no conflict of interest.
